# Plasma Soluble Prion Protein, a Potential Biomarker for Sport-Related Concussions: A Pilot Study

**DOI:** 10.1371/journal.pone.0117286

**Published:** 2015-02-02

**Authors:** Nam Pham, Hungbo Akonasu, Rhonda Shishkin, Changiz Taghibiglou

**Affiliations:** 1 Department of Pharmacology, College of Medicine, University of Saskatchewan, Saskatoon, Canada; 2 College of Kinesiology and Huskies Athletics, University of Saskatchewan, Saskatoon, Canada; Scuola Internazionale Superiore di Studi Avanzati, ITALY

## Abstract

Sport-related mild traumatic brain injury (mTBI) or concussion is a significant health concern to athletes with potential long-term consequences. The diagnosis of sport concussion and return to sport decision making is one of the greatest challenges facing health care clinicians working in sports. Blood biomarkers have recently demonstrated their potential in assisting the detection of brain injury particularly, in those cases with no obvious physical injury. We have recently discovered plasma soluble cellular prion protein (PrP^C^) as a potential reliable biomarker for blast induced TBI (bTBI) in a rodent animal model. In order to explore the application of this novel TBI biomarker to sport-related concussion, we conducted a pilot study at the University of Saskatchewan (U of S) by recruiting athlete and non-athlete 18 to 30 year-old students. Using a modified quantitative ELISA method, we first established normal values for the plasma soluble PrP^C^ in male and female students. The measured plasma soluble PrP^C^ in confirmed concussion cases demonstrated a significant elevation of this analyte in post-concussion samples. Data collected from our pilot study indicates that the plasma soluble PrP^C^ is a potential biomarker for sport-related concussion, which may be further developed into a clinical diagnostic tool to assist clinicians in the assessment of sport concussion and return-to-play decision making.

## Introduction

Concussion is a complex pathophysiological process and is considered as a subset of mild traumatic brain injury (mTBI). It causes a transient disturbance of brain function resulting in less severe brain injury. Concussions are the consequence of a direct or indirect blow that results in a sudden angular acceleration or deceleration of the brain tissue within the calvarium. In the US alone, 3.8 million cases of sport-related concussions occur annually and high-contact sports such as American football, hockey, rugby, soccer, and basketball have among the highest incidence of concussion [1–4]. Considering unreported cases, it is highly likely that the incidence of sport-related concussions is even higher [5].

Clinical manifestations of sport-related concussions may include a variety of symptoms such as loss of consciousness, headache, dizziness, amnesia, nausea, confusion, fatigue, sleep disturbances, balance and memory impairment, slurred speech, and light sensitivity. At the molecular pathophysiological levels, most of these symptoms are direct or indirect results of significant alterations in ionic balance, neurotransmitter activation, axonal integrity, and energy metabolism in the CNS [6,7].

Most sport-related concussions are benign and athletes typically recover within 7–10 days or even longer if given adequate rest and appropriate therapy. During this sensitive period of recovery, individuals are extremely at risk should they suffer a subsequent head injury. Multiple concussions within a short period of time may lead to devastating long-term sequelae and prolonged functional impairment, including post-concussive syndrome, neurodegenerative diseases, chronic traumatic encephalopathy, as well as rare catastrophic consequences called second impact syndrome [8–10]. Second impact syndrome is a post-concussion cerebral edema, which results in coma and severe neurological deficits and is often deadly. Thus, it is absolutely essential to manage concussions properly and to avoid repetitive concussive events in those who have already experienced mTBI. Since most mTBI cases show no abnormalities on computed tomography (CT) and conventional magnetic resonance imaging (MRI), identifying those athletes affected by concussion remains a challenging issue for health care clinicians [11]. A promising approach to ease these challenges has focused on the detection of protein biomarkers of sport-related concussion. Protein biomarkers are readily accessible in biological fluids such as plasma and serum, which may serve as valuable tools in identifying concussed athletes at greater risk for deterioration and in the guidance of immediate post-concussion therapeutic interventions as well as decision making on return-to-play. Several potential protein biomarkers have been identified for TBI, of which a few have been tested in sport related concussion (reviewed in [12–18]). Among these potential protein biomarkers, S100B, cleaved tau (C-tau), glial fibrillary acidic protein (GFAP), neuron-specific enolase (NSE), Myelin-basic protein (MBP), Ubiquitin C-terminal hydrolase-L1*(*UCH-L1), **α**II-spectrin breakdown products (SBDPs), Interleukin-6 (IL-6) and tumor necrosis factor-alpha (TNF- α) have been more widely studied (reviewed in [17,18]). However, to our knowledge there are no reports indicating cellular prion protein (PrP^C^) as a potential biomarker of mTBI/concussion. The present pilot study seeks to address this question as to whether assessment of plasma PrP^C^ may also be indicative of sport concussion.

PrP^C^ is a ubiquitous glycoprotein distributed throughout many cell types and tissues in mammals with the highest expression levels within the central nervous system (CNS) (reviewed in [19,20]). This 208–209 amino acids long protein is almost entirely located within the extracellular domain on dynamic lipid raft compartments of the plasma membrane (PM) tethered by a glycosyl-phosphatidyl-inositol (GPI) anchor [19,20]. Various physiological functions are attributed to PrP^C^ in CNS including cellular adhesion, cell signaling, ion homeostasis, and neuroprotection [19,20]. Because of the PrP^C^’s extracellular orientation, it is possible that during a concussive event, linear and/or rotational forces transmitted to the brain may cause the tenuously bound PrP^C^ to dislodge and collect within the systemic circulation. We have most recently shown in an animal model of blast exposure that plasma PrP^C^ is a potential biomarker for determining TBI [21]. Other studies have also investigated PrP^C^ as a biomarker for different pathologic states associated with neuronal damage [22–26], but none have examined its potential association with conventional head injury. Additionally, a previous study has shown in an animal model that following 24 hours from blast exposure, the prion protein gene (PRNP) is one of many upregulated genes within the brain, but it was not further examined according to the study design [27]. It is therefore possible that similar to blast TBI models, PrP^C^ can be used as a biomarker for sports-related mTBI as well. In this pilot study, we addressed this hypothesis by collecting blood plasma from the normal healthy university student population (age 18–30 years old) as well as concussed student athletes for quantification of PrP^C^. In this pilot study, we identified plasma PrP^C^ as a potential biomarker for sport-related concussions.

## Material and Methods

### Athletes and non-athletes recruitment

The study was approved by the Biomedical Research Ethics Committee of the University of Saskatchewan, Canada (Bio # 13–195). Members of University of Saskatchewan Huskies Athletic teams including Canadian football, ice hockey, basketball, and soccer teams as well as healthy non-athlete male and female university students were asked to participate in the investigation. Individuals were asked in a questionnaire whether they were in good standing health without any existing illnesses or condition and whether they had recently (<6 months) suffered a head injury. Those who were not well or had suffered an injury were excluded from the study. Altogether, participants of high-contact sports were recruited as follows: ice hockey (n = 17), football (n = 20), soccer (n = 4), basketball (n = 18), and wrestling (n = 6). Samples were also collected from athletes in typically low contact sports such as volleyball and cross country (n = 11). For normal values, 27 additional samples were collected from the non-athlete university student population. In total six concussed athletes were identified using the sports concussion assessment tool (SCAT3) concussion assessment criteria [28] and their post-concussion blood samples collected 1–7 day post-incidence depending on subjects availability. For the summary characteristics of participants involved in this study see Table 1.

**Table 1:** Study participants’ description.

Participant Summary							
		Age (years)			PrP^C^ Concentration (ng/mL)		
	n	Mean ± SD	Median	Range	Mean ± SEM	Median	Range
**Non-Athlete**	**27**	**24.48 ± 2.99**	**24.00**	**18–30**	**2.02 ± 0.15**	**2.23**	**0.72–3.87**
*Male*	15	24.67 ± 1.76	24.00	22–29	2.12 ± 0.18	2.32	1.11–3.41
*Female*	12	24.25 ± 4.14	23.50	18–30	1.89 ± 0.27	2.27	0.72–3.87
**Athlete**	**76**	**20.04 ± 1.84**	**20.00**	**18–26**	**1.59 ± 0.64**	**1.51**	**0.56–3.66**
*Male*	39	20.41 ± 1.92	20.00	18–24	1.44 ± 0.10	1.34	0.56–3.17
*Female*	37	19.65 ± 1.70	19.00	18–26	1.75 ± 0.10	1.59	1.05–3.66
**Combined**	**103**	**21.20 ± 2.94**	**21.00**	**18–30**	**1.70 ± 0.07**	**1.55**	**0.56–3.87**
*Male*	54	21.59 ± 2.67	22.00	18–29	1.63 ± 0.10	1.40	0.56–3.41
*Female*	49	20.78 ± 3.18	20.00	18–30	1.79 ± 0.10	1.62	0.72–3.87

Summary of participants’ (non-athlete, athlete, and combined) age and plasma PrP^C^ concentration stratified by gender.

SD = Standard Deviation

SEM = Standard Error of the Mean

### Plasma Separation

All sample collection was performed following signed and informed consent prior to invasive procedure and sample testing as outlined by the TCPS2. Samples were alphanumerically coded and sample testing was performed single blinded. A small sample of venous blood (2 mL) is collected from both athletes and non-athletes into lithium heparin coated vacutainer tubes (BD vacutainer PST, #367962). Samples are immediately placed on ice to allow stable transport to laboratory setting. Samples were then centrifuged at 10,000G for 10 minutes for plasma isolation. Plasma fraction was aliquoted and immediately stored at-80^°^C for future analysis.

### Plasma PrP^C^ ELISA

For sensitive quantification of full-length soluble PrP^C^, we employed an ELISA technique using a commercially available qualitative assay kit (Spi Bio A05201, Paris, FR) and modified the manufacturer’s protocol to allow sensitive and accurate quantification as previously employed [21]. Pure full-length recombinant PrP^C^ (Prionatis, α-Rec Mouse PrP-RPA0101S, Zurich, CH) was used for producing serial dilutions (0.625–20 ng/mL) in order to establish the calibration curve for quantifying samples. All samples and PrP^C^ protein standards were diluted in the manufacturer’s provided dilution buffer solution (1 M phosphate, 1% BSA, 4 M NaCl, 10mM EDTA, and 0.1% sodium azide). Remaining solutions and reagents provided by the manufacturer were reconstituted and prepared according to the suggested protocol. Briefly, overall protein concentration of individual samples was first determined in triplicate using the Bio-Rad DC protein assay (Sigma-Aldrich, bovine albumin, A-9647, Oakville ON). Samples and standards were loaded in equal volume in triplicate in the kit’s 96 microwell plate strips. Diluted samples were loaded as such that each well contained approximately overall protein amounts of 75–100 μg. The plate was then incubated overnight at 4°C with shaking to allow adequate antigen binding to well-embedded monoclonal antibodies, specific to the 144–153 amino acid sequence within the C-terminus. After rigorous washing (4M phosphate, pH 7.4), the wells are incubated with an acetylcholinesterase- (AChE) Fab’ conjugated antibody solution, targeting the octorepeat N-terminus region, for two hours at RT with shaking, thus completing a double-antibody sandwich. After another cycle of rigorous washing, Ellman’s reagent was added in equal volume to each well, and incubated in the dark for 30 minutes at RT with shaking. Any immobilized AChE-conjugated antibody bound to PrP^C^ therefore reacts with Ellman’s reagent to produce a colorimetric reaction in solution proportional to the concentration of PrP^C^, which is read using a microplate reader at 405nm (Molecular Devices, LLC., SpectraMax M5, Sunnyvale CA, USA). Raw absorbance values were interpolated along the standard calibration curve and converted into PrP^C^ concentration values.

### Western blotting

**Fig 1:**
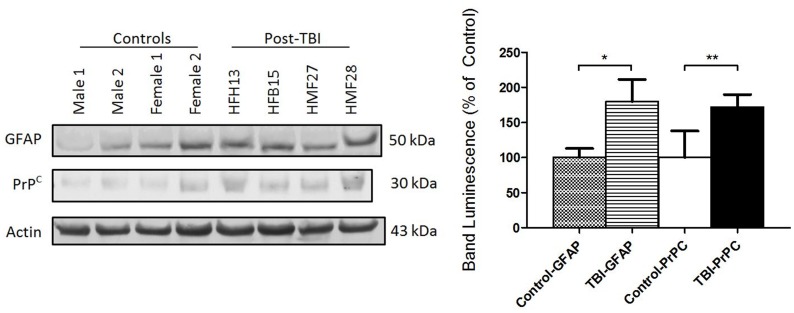
Western blot for protein biomarkers. Semi-quantitative band density analysis determined that both GFAP and PrP^C^ protein content were significantly elevated in post-TBI samples (n = 4) in comparison to randomly selected controls (n = 8). All bands were normalized to housekeeping protein actin. Mean post-TBI GFAP band intensity was higher (180.0% ± 31.4% SEM) than in controls (100% ± 13.0%) (two-tailed t-test, *p<0*.*034*). Mean post-TBI PrP^C^ band intensity was also higher (171.9% ± 18.0%) than in controls (100.0% ± 11.0%) (two-tailed t-test, *p<0*.*0025*).

Western blotting was performed as previously described [21,29]. Protein concentration of plasma samples was determined using the Bio-Rad DC assay and 30μg protein per well was loaded into 15% acrylamide gels for SDS-PAGE. Protein was then transferred onto PVDF membrane (FluoroTrans, Pall Life Sciences) at 100V for 1 hour. Membranes were blocked in 5% bovine serum albumin in PBS-Tween 20 (0.1%) at room temperature (RT) for 1 hour. Primary antibodies used for immunoblotting targeted PrP^C^ (Santa Cruz sc-7693, 1:500), GFAP (Santa Cruz sc-6170, 1:500), and actin (Santa Cruz sc-1616, 1:500). Primary antibody incubation was performed either at room temperature for 1–2 hours or at 4°C overnight. Following stringent washing and secondary antibody incubation steps, membranes were exposed to enhanced chemiluminescence reagent (Amersham) and exposed to x-ray film. Protein bands of interest were analyzed using NIH ImageJ software and normalized to that of the actin loading control in each sample lane. Control samples selected for Western blotting includes baseline (n = 3) and randomly selected athlete baseline or normal controls (n = 5). A representative blot is provided below (see Fig. 1).

### Statistical Analysis

Statistical analysis for all data was performed using Graphpad Prism 5 statistical package. Student’s *T*-test for statistical significance was performed for plasma PrP^C^ mean value comparison of the following groupings: male vs. female, athletes vs. non-athletes, and post-TBI vs. baseline or combined athletes and non-athletes (representative of the general population). One-way analysis of variance (one-way ANOVA) was used to determine whether there is significant variation of mean PrP^C^ concentration among different age groups. Results were considered statistically significant when *p* ≤ 0.05.

## Results

### Plasma levels of soluble cellular prion protein levels in healthy young male and female adults

**Fig 2:**
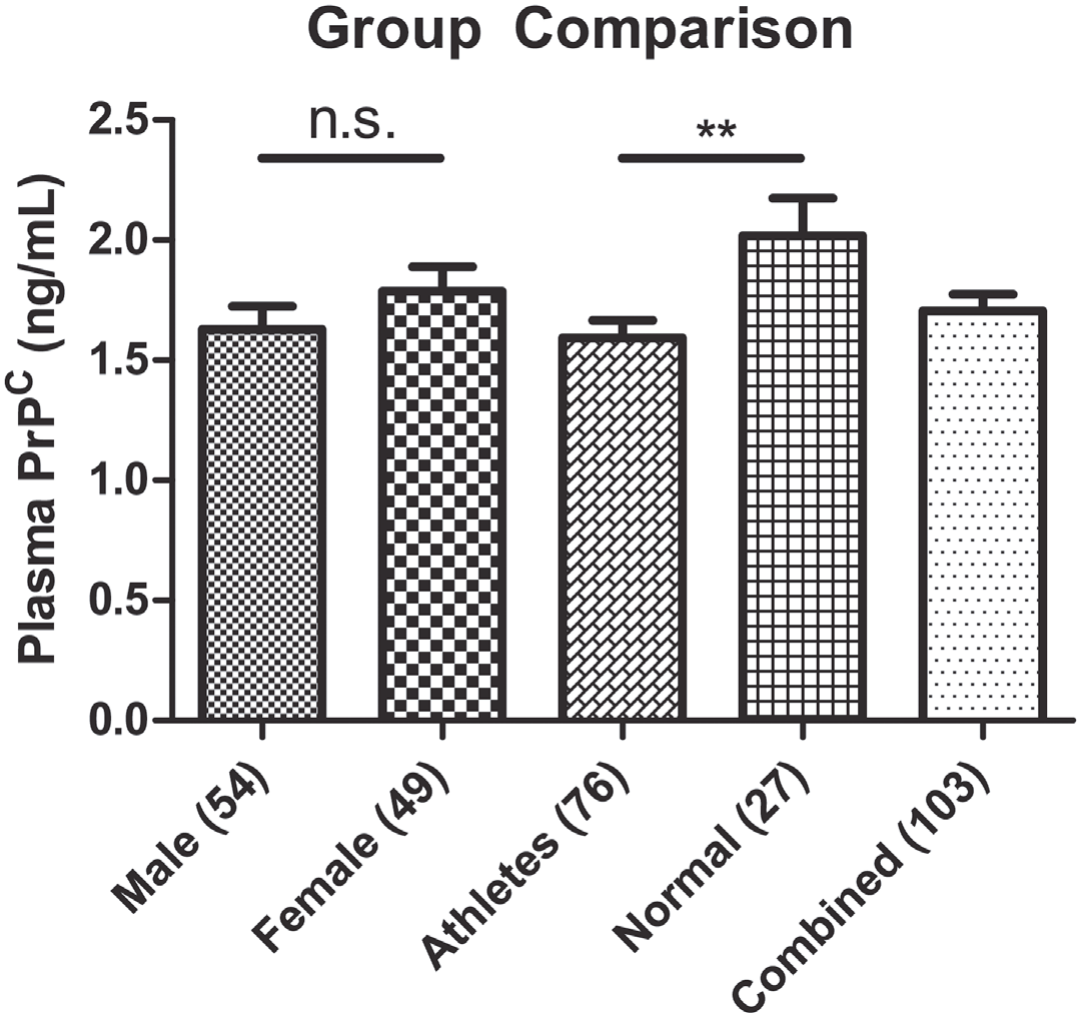
Group Comparison of Plasma PrP^C^. Two-tailed unpaired student’s t-test shows no significant difference between male (n = 54; 1.63 ng/mL **±** 0.10 SEM) and female (n = 49; 1.79 ng/mL **±** 0.10 SEM) (*p>0*.*05*). *T* test of athletes (n = 76, 1.59 ng/mL **±** 0.07 SEM) vs. the normal non-athlete population (n = 27; 2.02 ng/mL **±** 0.15 SEM) shows significant difference between mean PrP^C^ concentrations (*p<0*.*01*).

In order to investigate the possibility that the plasma level of PrP^C^ rises following mTBI, we first measured normal soluble PrP^C^ levels in the general population aged 18 years and above without significant confounds due to illness, health condition, or concussion within the past six months. T-test comparison between male (mean ± SEM = 1.63 ng/mL ± 0.10, n = 54) vs. female (1.79 ng/mL ± 0.10, n = 49) showed no significant difference in mean concentration of plasma PrP^C^ (*p = 0*.*2578*) (see Fig. 2). Additionally, we found a slight significant difference in mean plasma PrP^C^ between off season athletes’ baselines (1.59 ± 0.073, n = 76) vs. normal non-athlete students (2.012 ± 0.15, n = 27) (*p = 0*.*0065*) (see Fig. 2).

**Fig 3:**
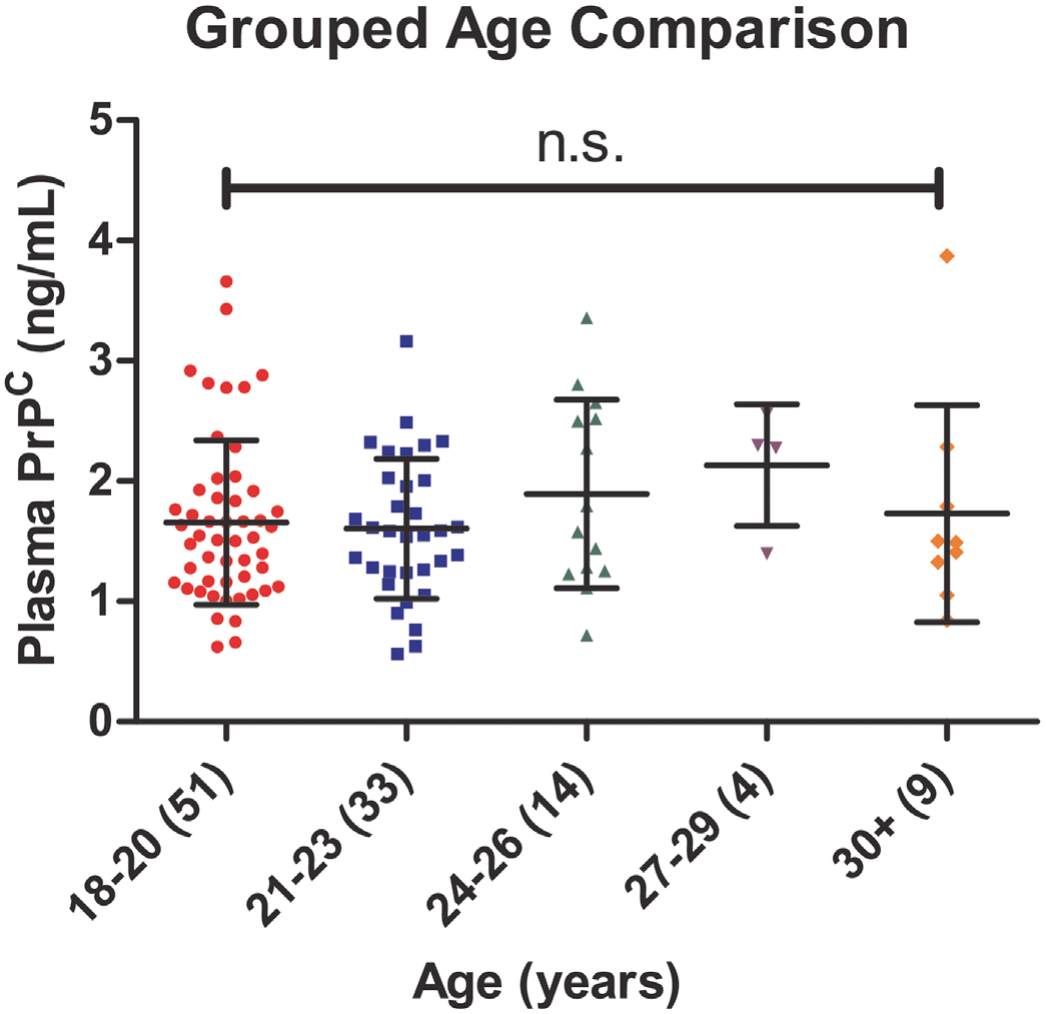
Comparison of Plasma PrP^C^ with Age. One-way ANOVA of PrP^C^ concentrations for different age groups shows there is no significant difference between mean concentrations for subjects between the ages of 18–20 (n = 51; 1.66 ng/mL ± 0.68 SD), 21–33 (n = 33; 1.61 ng/L ± 0.58 SD), 24–26 (n = 14; 1.89 ng/mL ± 0.78 SD), 27–29 (n = 4; 2.13 ng/mL ± 0.51 SD), and those 30 and over (n = 9; 1.73 ng/mL ± 0.90 SD) (*p = 0*.*4702*).

Furthermore, aggregate results were separated into five age groups to determine any significant difference in plasma PrP^C^ across different age brackets (see Fig. 3). One-way ANOVA for determining variation between mean plasma PrP^C^ concentration across age groups showed no significant difference across the different age groups (*p = 0*.*4702*).

### Plasma soluble PrP^C^ level increases in concussed athletes

**Fig 4:**
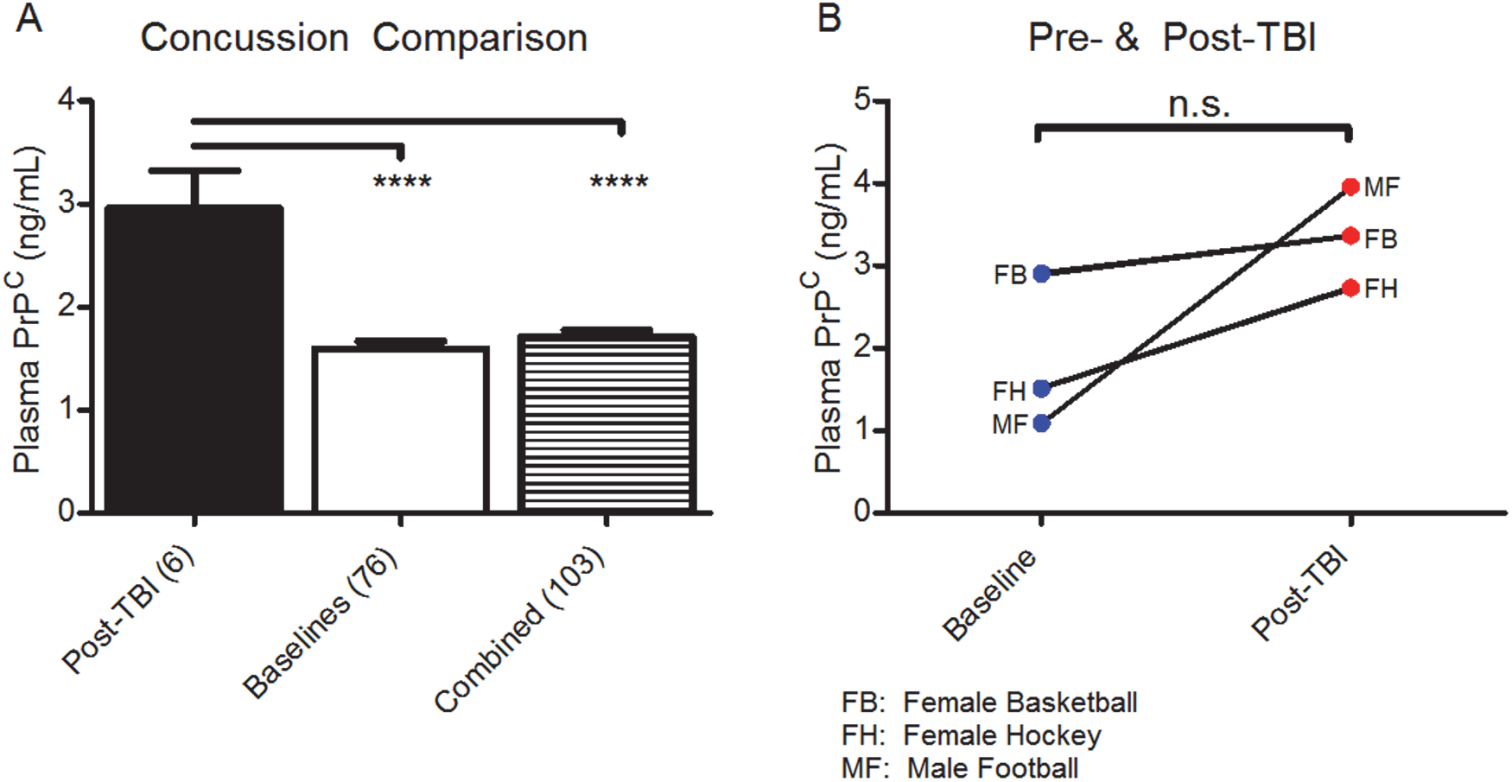
Comparison between Normal and Post-Concussion. **A)** Two-tailed unpaired student’s t-test shows post-TBI PrP^C^ concentrations (n = 6; 2.96 ng/mL **±** 0.37 SEM) are significantly elevated compared with either offseason athlete baseline concentration (n = 76’ 1.59 ng/mL **±** 0.07 SEM)(*p< 0*.*0001*), or both athletes and non-athletes combined (n = 103; 1.70 ng/mL **±** 0.07 SEM)(*p<0*.*0001*). **B)** Two-tailed paired t-test shows there was no significant difference between three sets of pre- and post-TBI PrP^C^ values (*p = 0*.*1666*).

During the 2013–2014 season, the Huskies Athletic teams had 4 female and 2 male participants within the study whom suffered injuries causing concussion while engaging in various sports including Canadian football, ice hockey, basketball and wrestling. Initial signs and symptoms following injury were evaluated for the six athletes by clinician administered cognitive testing along with self-reporting of symptoms and their severity along a scale using the SCAT3 criteria (see Table 2). For virtually all incidents, the injuries sustained by the athletes involved a significant blow to the head and/or included rapid whiplash acceleration of the head along the neck. Depending on access and appropriate convenience to the concussed athletes, their blood samples were collected within 24hrs to 7 days post-mTBI. Comparison of mean plasma PrP^C^ in post-concussion samples (2.96 ng/mL ± 0.37, n = 6) was found to be significantly higher (*p<0*.*0001*) than levels in baseline samples collected in the offseason (1.59 ng/mL **±** 0.07, n = 76) and against combined baselines with the normal population (1.70 ng/mL **±** 0.07, n = 103) (see Fig. 4-A). Of the 76 baseline samples collected from athlete participants during the offseason, only three individuals sustained a concussion during the season to allow pre- and post-TBI comparison (see Fig. 4-B). Paired t-test comparison shows there was no significant difference between three sets of pre- and post-TBI PrP^C^ values (*p = 0*.*1666*). Confirmatory immunoblotting was performed comparing post-TBI samples against combined baseline and non-athlete controls showing that there is a significant increase in PrP^C^ (*p = 0*.0025) and an additional marker of TBI in GFAP (*p = 0*.*034*).

**Table 2:** Summary of participants’ head injury.

#ID	Method of Injury	Initial Signs & Symptoms				
		LOC	Balance	Cognitive	Symptom Scale (#symptoms/total score)	Time from Injury to Collection
HMF27	Helmet to helmet contact	No	Yes	Yes	21/81	1 day
HMF28	Unknown	No	No	No	16/46	5 days
HFB15	Elbow to head	No	No	Yes	14/39	6 days
HFH13	Collision with player head on but no head contact, whiplash	No	Yes	No	6/13	2 days
HFW1	Punch to face	No	No	No	12/23	7 days
HFW2	Knee to temple, punch to head	No	Yes	No	19/71	7 days

Description and sideline assessment of injured athletes performed by team clinician. Athletes’ self-assessed symptoms and severity provided as calculated from the SCAT3 assessment criteria. Period of time between injury and blood sample collection is also noted.

LOC = Loss of Consciousness

## Discussion

Sport-related concussions are the most common cases of mTBI among children and young adults [30–32]. Despite several clinical symptoms and manifestations, it is believed that the majority of concussive events still remain unreported or ignored. Considering limitations and shortcomings of diagnostic medical imaging techniques, it is thus necessary to have access to more reliable and easy to use quantitative diagnostic concussion test to identify concussive athletes and to reduce the risk of potential catastrophic second impact syndrome. Protein biomarkers in biological fluids have opened new horizons in TBI and concussion diagnosis. In the present pilot study, we examined concentrations of plasma soluble PrP^C^ in university student athletes who had a sports-related concussion (six concussion cases in the last season). We found that the post-concussion levels of plasma soluble PrP^C^ were significantly higher when compared with the normal plasma PrP^C^ values in young adults.

PrP^C^ is a loosely associated lipid raft protein known for several important physiological functions including its neuroprotective role in the brain. In this pilot study we hypothesized, that in a concussive event, the applied force on the brain may dislodge PrP^C^ off its neuronal lipid rafts and allow the protein to collect within the CSF and systemic circulation. Therefore, the plasma soluble PrP^C^ could be used as a potential biomarker for mTBI diagnosis. Although PrP^C^ is prominently expressed in CNS, emerging evidence indicates that soluble PrP^C^ could cross the blood brain barrier in a bidirectional manner [33]. Additionally, the BBB may be disrupted in a concussive event [34], allowing for even more pooling of PrP^C^ within the bloodstream. PrP^C^ levels in biological fluids such as cerebral spinal fluid (CSF) and plasma have been previously used as a useful biomarker for certain pathological conditions [22,24–26,35–37]. Increased plasma PrP^C^ concentrations have been reported following stroke and in patients with various neurodegenerative diseases [22,26]. More recent studies have shown that patients with cerebrovascular disease or vascular endothelial damage had higher levels of plasma PrP^C^ than control values [25,38,39]. Moreover, the role of soluble PrP^C^, in the modulation of immune cell activation centrally and peripherally, was proposed to be used as a biomarker for neuroinflammation and encephalitis; particularly in cases related to HIV-infected individuals [24]. To our best knowledge, the soluble plasma PrP^C^ has not been studied as a potential biomarker for the concussion.

Most of the studies mentioned above were conducted with older subjects, whereas our subjects are primarily young adults who are mostly involved in high contact sports. Thus a challenge of this study was to establish normal values and determine whether there is any significant variance due to age or gender. Age-dependent expression of PrP^C^ has been previously reported [40], but we did not observe any significant difference across various age groups (see Fig. 3). We must note that in total 111 participants donated a blood sample for this study, but eight were excluded from the pool of controls due to their age. We also observed a slightly higher trend, though non-significant, concentration of soluble PrP^C^ in female plasma samples when compared with males. This is in line with observations previously reported [41]. Interestingly, lower soluble PrP^C^ level was observed among offseason athlete baseline values as compared with controls (non-athlete students). A possible explanation for this is that collegiate level athletes are in exceptional physical condition, thus having lower blood pressure compared to non-athletes. Since PrP^C^ is also expressed on endothelial cell lining, those with higher blood pressure may release more PrP^C^ into the blood, a trend which was observed in a hypertensive population [38,41]. However, due to unequal sample sizing (76 baseline vs. 27 non-athletes), we cannot rule out the possibility that heterogeneity of results from the normal sample group being more pronounced. The soluble PrP^C^ is also involved in activation of immune cells and immune response [42,43]. We thus speculate that the lower soluble PrP^C^ levels in off-season athletes may be required to accommodate relatively lower pro-inflammatory cytokines condition necessary for promoting off-seasonal CNS repair, although more studies are needed to support this notion.

According to our pilot study results, we found using Western blotting and ELISA that there is indeed a significant rise of the plasma soluble PrP^C^ in post-mTBI/concussion samples compared with both the control adult population and offseason athlete plasma samples (see Fig. 4A). Additionally, Western blotting confirmed elevated plasma levels of GFAP in post-TBI samples compared to controls, thus indicating the presence of injury (see Fig. 1) [44]. Altogether, we can state that elevated plasma PrP^C^ is associated with sport-related concussion. However, we must also report that comparison between pre- and post-TBI values show no significant difference (see Fig. 4B). We must stress that not all athletes submitted a baseline sample during the offseason. Therefore, of the six concussion samples collected we were limited to only three corresponding baselines to compare against, but these pairs do show an upward trend in plasma PrP^C^ concentration despite delayed periods in collection following injury (1–6 days). Extreme examples of this can be seen when comparing the trend for MF and FB samples. The MF (male football) post-injury sample was collected shortly after the injury (1 day) while the FB (female basketball) was collected much later (6 days). It is safe to assume that the lack of any appreciable elevation in PrP^C^ within the FB samples is a result of this time latency. Another factor to consider is that of all the injured athletes sampled, the MF participant had the highest number of self-reported symptoms (21) with high severity (81 out of possible 126 symptom score) (see table 2). Therefore higher injury severity may contribute towards higher plasma PrP^C^ following injury in the MF participant. Unfortunately, regression and correlation analysis of PrP^C^ levels to injury severity could not be performed in this study due to non-controlled sampling times following injury, but future exploration in this aspect would strengthen the feasibility of PrP^C^ as an ideal biomarker for TBI. Due to the limitation in the number of concussed athletes and variation in the time period between injury and collection, which ranged between 1 and 7 days, we cannot definitively determine whether this rise in plasma PrP^C^ is directly attributed to protein shedding from the CNS, circulatory blood cells, or even from upregulated PrP^C^ protein expression [27]. Recent evidence suggests that normal PrP^C^ may be also secreted out (on exosomes) from cultured neurons following toxic challenges such as NMDA-induced excitotoxicity [45]. Moreover, PrP^C^ containing exosomes have recently been isolated from human plasma [46]. Since neuronal excitotoxicity plays a major role in the pathogenesis of TBI (reviewed in [47,48]), it is thus plausible to hypothesize that the CNS is a major contributor to plasma soluble PrP^C^ following a concussion.

The goal of this study was to determine the feasibility of using plasma levels of PrP^C^ in athletes following concussion to be compared against controls as a biomarker for sports concussion. Results obtained from gender and different age groups of young adults show no significant difference which means there is no gender and age variation in human plasma PrP^C^ concentration, making it an ideal parameter for testing as a biomarker. Although more investigation with increased sample size (particularly concussion samples) is needed to solidify our observation and finding, the results presented in this pilot study provide first evidence that easily accessible plasma soluble PrP^C^ might have a relevant association with sport-related concussion/mTBI and potentially be a useful biomarker to identify concussive athletes at risk.

## References

[1] Daneshvar DH, Nowinski CJ, McKee AC, Cantu RC (2011) The epidemiology of sport-related concussion. Clin Sports Med 30: 1–17, vii 10.1016/j.csm.2010.08.006 21074078 PMC2987636

[2] Harmon KG, Drezner J, Gammons M, Guskiewicz K, Halstead M, et al (2013) American Medical Society for Sports Medicine position statement: concussion in sport. Clin J Sport Med 23: 1–18. 10.1097/JSM.0b013e31827f5f93 23269325

[3] Langlois JA, Rutland-Brown W, Wald MM (2006) The epidemiology and impact of traumatic brain injury: a brief overview. J Head Trauma Rehabil 21: 375–378. 16983222 10.1097/00001199-200609000-00001

[4] Meehan WP 3rd, Micheli LJ (2011) Concussion results in deficits in neurocognitive functioning. Preface. Clin Sports Med 30: xvii–iii. 10.1016/j.csm.2010.09.008 21074077

[5] Meehan WP 3rd, Mannix RC, O’Brien MJ, Collins MW (2013) The prevalence of undiagnosed concussions in athletes. Clin J Sport Med 23: 339–342. 10.1097/JSM.0b013e318291d3b3 23727697 PMC3758800

[6] Barkhoudarian G, Hovda DA, Giza CC (2011) The molecular pathophysiology of concussive brain injury. Clin Sports Med 30: 33–48, vii–iii. 10.1016/j.csm.2010.09.001 21074080

[7] McKee AC, Daneshvar DH, Alvarez VE, Stein TD (2014) The neuropathology of sport. Acta Neuropathol 127: 29–51. 10.1007/s00401-013-1230-6 24366527 PMC4255282

[8] Boden BP, Tacchetti RL, Cantu RC, Knowles SB, Mueller FO (2007) Catastrophic head injuries in high school and college football players. Am J Sports Med 35: 1075–1081. 17351124 10.1177/0363546507299239

[9] Gavett BE, Stern RA, McKee AC (2011) Chronic traumatic encephalopathy: a potential late effect of sport-related concussive and subconcussive head trauma. Clin Sports Med 30: 179–188, xi 10.1016/j.csm.2010.09.007 21074091 PMC2995699

[10] Halstead ME, Walter KD (2010) American Academy of Pediatrics. Clinical report—sport-related concussion in children and adolescents. Pediatrics 126: 597–615. 10.1542/peds.2010-2005 20805152

[11] Belanger HG, Vanderploeg RD, Curtiss G, Warden DL (2007) Recent neuroimaging techniques in mild traumatic brain injury. J Neuropsychiatry Clin Neurosci 19: 5–20. 17308222 10.1176/jnp.2007.19.1.5

[12] Forde CT, Karri SK, Young AM, Ogilvy CS (2014) Predictive markers in traumatic brain injury: opportunities for a serum biosignature. Br J Neurosurg 28: 8–15. 10.3109/02688697.2013.815317 23855389

[13] Guingab-Cagmat JD, Cagmat EB, Hayes RL, Anagli J (2013) Integration of proteomics, bioinformatics, and systems biology in traumatic brain injury biomarker discovery. Front Neurol 4: 61 10.3389/fneur.2013.00061 23750150 PMC3668328

[14] Jeter CB, Hergenroeder GW, Hylin MJ, Redell JB, Moore AN, et al (2013) Biomarkers for the diagnosis and prognosis of mild traumatic brain injury/concussion. J Neurotrauma 30: 657–670. 10.1089/neu.2012.2439 23062081

[15] Strathmann FG, Schulte S, Goerl K, Petron DJ (2014) Blood-based biomarkers for traumatic brain injury: Evaluation of research approaches, available methods and potential utility from the clinician and clinical laboratory perspectives. Clin Biochem. 10.1016/j.clinbiochem.2014.12.017 24486649

[16] Wolf H, Frantal S, Pajenda GS, Salameh O, Widhalm H, et al (2013) Predictive value of neuromarkers supported by a set of clinical criteria in patients with mild traumatic brain injury: S100B protein and neuron-specific enolase on trial: clinical article. J Neurosurg 118: 1298–1303. 10.3171/2013.1.JNS121181 23451906

[17] Yokobori S, Hosein K, Burks S, Sharma I, Gajavelli S, et al (2013) Biomarkers for the clinical differential diagnosis in traumatic brain injury—a systematic review. CNS Neurosci Ther 19: 556–565. 10.1111/cns.12127 23710877 PMC6493562

[18] Zetterberg H, Smith DH, Blennow K (2013) Biomarkers of mild traumatic brain injury in cerebrospinal fluid and blood. Nat Rev Neurol 9: 201–210. 10.1038/nrneurol.2013.9 23399646 PMC4513656

[19] Aguzzi A, Calella AM (2009) Prions: protein aggregation and infectious diseases. Physiol Rev 89: 1105–1152. 10.1152/physrev.00006.2009 19789378

[20] Yusa S, Oliveira-Martins JB, Sugita-Konishi Y, Kikuchi Y (2012) Cellular prion protein: from physiology to pathology. Viruses 4: 3109–3131. 10.3390/v4113109 23202518 PMC3509686

[21] Pham N, Sawyer T, Wang Y, Rastgar Jazii F, Vair C, et al (2015) Primary Blast-induced Traumatic Brain Injury in Rats Leads to Increased Prion Protein in Plasma: A Potential Biomarker for Blast-Induced Traumatic Brain Injury. J Neurotrauma 32: 58–65. 10.1089/neu.2014.3471 25058115 PMC4273182

[22] Mitsios N, Saka M, Krupinski J, Pennucci R, Sanfeliu C, et al (2007) Cellular prion protein is increased in the plasma and peri-infarcted brain tissue after acute stroke. J Neurosci Res 85: 602–611. 17149767 10.1002/jnr.21142

[23] Megra B, Eugenin E, Roberts T, Morgello S, Berman JW (2013) Protease resistant protein cellular isoform (PrP(c)) as a biomarker: clues into the pathogenesis of HAND. J Neuroimmune Pharmacol 8: 1159–1166. 10.1007/s11481-013-9458-4 23616272 PMC3797864

[24] Roberts TK, Eugenin EA, Morgello S, Clements JE, Zink MC, et al (2010) PrPC, the cellular isoform of the human prion protein, is a novel biomarker of HIV-associated neurocognitive impairment and mediates neuroinflammation. Am J Pathol 177: 1848–1860. 10.2353/ajpath.2010.091006 20724601 PMC2947280

[25] Krupinski J, Turu MM, Luque A, Badimon L, Slevin M (2008) Increased PrPC expression correlates with endoglin (CD105) positive microvessels in advanced carotid lesions. Acta Neuropathol 116: 537–545. 10.1007/s00401-008-0427-6 18810471

[26] Volkel D, Zimmermann K, Zerr I, Bodemer M, Lindner T, et al (2001) Immunochemical determination of cellular prion protein in plasma from healthy subjects and patients with sporadic CJD or other neurologic diseases. Transfusion 41: 441–448. 11316892 10.1046/j.1537-2995.2001.41040441.x

[27] Kochanek PM, Dixon CE, Shellington DK, Shin SS, Bayir H, et al (2013) Screening of biochemical and molecular mechanisms of secondary injury and repair in the brain after experimental blast-induced traumatic brain injury in rats. J Neurotrauma 30: 920–937. 10.1089/neu.2013.2862 23496248 PMC5586163

[28] Guskiewicz KM, Register-Mihalik J, McCrory P, McCrea M, Johnston K, et al (2013) Evidence-based approach to revising the SCAT2: introducing the SCAT3. Br J Sports Med 47: 289–293. 10.1136/bjsports-2013-092225 23479486

[29] Pham N, Dhar A, Khalaj S, Desai K, Taghibiglou C (2014) Down regulation of brain cellular prion protein in an animal model of insulin resistance: possible implication in increased prevalence of stroke in pre-diabetics/diabetics. Biochem Biophys Res Commun 448: 151–156. 10.1016/j.bbrc.2014.04.071 24780399

[30] Noble JM, Hesdorffer DC (2013) Sport-related concussions: a review of epidemiology, challenges in diagnosis, and potential risk factors. Neuropsychol Rev 23: 273–284. 10.1007/s11065-013-9239-0 24242889

[31] Selassie AW, Wilson DA, Pickelsimer EE, Voronca DC, Williams NR, et al (2013) Incidence of sport-related traumatic brain injury and risk factors of severity: a population-based epidemiologic study. Ann Epidemiol 23: 750–756. 10.1016/j.annepidem.2013.07.022 24060276 PMC4021712

[32] Stewart TC, Gilliland J, Fraser DD (2013) An epidemiologic profile of pediatric concussions: identifying urban and rural differences. J Trauma Acute Care Surg 76: 736–742. 10.1097/TA.0b013e3182aafdf5 24553542

[33] Banks WA, Robinson SM, Diaz-Espinoza R, Urayama A, Soto C (2009) Transport of prion protein across the blood-brain barrier. Exp Neurol 218: 162–167. 10.1016/j.expneurol.2009.04.025 19422824 PMC2806677

[34] Marchi N, Bazarian JJ, Puvenna V, Janigro M, Ghosh C, et al (2013) Consequences of repeated blood-brain barrier disruption in football players. PLoS One 8: e56805 10.1371/journal.pone.0056805 23483891 PMC3590196

[35] Meyne F, Gloeckner SF, Ciesielczyk B, Heinemann U, Krasnianski A, et al (2009) Total prion protein levels in the cerebrospinal fluid are reduced in patients with various neurological disorders. J Alzheimers Dis 17: 863–873. 10.3233/JAD-2009-1110 19542614

[36] Picard-Hagen N, Gayrard V, Viguie C, Moudjou M, Imbs C, et al (2006) Prion protein in the cerebrospinal fluid of healthy and naturally scrapie-affected sheep. J Gen Virol 87: 3723–3727. 17098990 10.1099/vir.0.81859-0

[37] Torres M, Cartier L, Matamala JM, Hernandez N, Woehlbier U, et al (2012) Altered Prion protein expression pattern in CSF as a biomarker for Creutzfeldt-Jakob disease. PLoS One 7: e36159 10.1371/journal.pone.0036159 22558368 PMC3338608

[38] Simak J, Holada K, D’Agnillo F, Janota J, Vostal JG (2002) Cellular prion protein is expressed on endothelial cells and is released during apoptosis on membrane microparticles found in human plasma. Transfusion 42: 334–342. 11961239 10.1046/j.1537-2995.2002.00072.x

[39] Starke R, Drummond O, MacGregor I, Biggerstaff J, Gale R, et al (2002) The expression of prion protein by endothelial cells: a source of the plasma form of prion protein? Br J Haematol 119: 863–873. 12437673 10.1046/j.1365-2141.2002.03847.x

[40] Politopoulou G, Seebach JD, Schmugge M, Schwarz HP, Aguzzi A (2000) Age-related expression of the cellular prion protein in human peripheral blood leukocytes. Haematologica 85: 580–587. 10870113

[41] Breitling LP, Muller H, Stegmaier C, Kliegel M, Brenner H (2012) Association of prion protein with cognitive functioning in humans. Exp Gerontol 47: 919–924. 10.1016/j.exger.2012.08.001 22967749

[42] Haddon DJ, Hughes MR, Antignano F, Westaway D, Cashman NR, et al (2009) Prion protein expression and release by mast cells after activation. J Infect Dis 200: 827–831. 10.1086/605022 19642931

[43] Jeon JW, Park BC, Jung JG, Jang YS, Shin EC, et al (2013) The Soluble Form of the Cellular Prion Protein Enhances Phagocytic Activity and Cytokine Production by Human Monocytes Via Activation of ERK and NF-kappaB. Immune Netw 13: 148–156. 10.4110/in.2013.13.4.148 24009542 PMC3759712

[44] Metting Z, Wilczak N, Rodiger LA, Schaaf JM, van der Naalt J (2012) GFAP and S100B in the acute phase of mild traumatic brain injury. Neurology 78: 1428–1433. 10.1212/WNL.0b013e318253d5c7 22517109

[45] Wang KK, Zoltewicz JS, Chiu A, Zhang Z, Rubenstein R (2012) Release of Full-Length PrP(C) from Cultured Neurons Following Neurotoxic Challenges. Front Neurol 3: 147 10.3389/fneur.2012.00147 23093947 PMC3477638

[46] Ritchie AJ, Crawford DM, Ferguson DJ, Burthem J, Roberts DJ (2013) Normal prion protein is expressed on exosomes isolated from human plasma. Br J Haematol 163: 678–680. 10.1111/bjh.12543 24117007

[47] Algattas H, Huang JH (2014) Traumatic Brain Injury pathophysiology and treatments: early, intermediate, and late phases post-injury. Int J Mol Sci 15: 309–341. 10.3390/ijms15010309 24381049 PMC3907812

[48] Parsons MP, Raymond LA (2014) Extrasynaptic NMDA receptor involvement in central nervous system disorders. Neuron 82: 279–293. 10.1016/j.neuron.2014.03.030 24742457

